# Attribution of regional flood changes based on scaling fingerprints

**DOI:** 10.1002/2016WR019036

**Published:** 2016-07-10

**Authors:** Alberto Viglione, Bruno Merz, Nguyen Viet Dung, Juraj Parajka, Thomas Nester, Günter Blöschl

**Affiliations:** ^1^ Institute of Hydraulic Engineering and Water Resources ManagementVienna University of TechnologyViennaAustria; ^2^ Helmholtz Centre Potsdam, GFZ German Research Centre for GeosciencesPotsdamGermany

**Keywords:** flood change, Bayesian attribution, multiple driver attribution, scaling fingerprints

## Abstract

Changes in the river flood regime may be due to atmospheric processes (e.g., increasing precipitation), catchment processes (e.g., soil compaction associated with land use change), and river system processes (e.g., loss of retention volume in the floodplains). This paper proposes a new framework for attributing flood changes to these drivers based on a regional analysis. We exploit the scaling characteristics (i.e., fingerprints) with catchment area of the effects of the drivers on flood changes. The estimation of their relative contributions is framed in Bayesian terms. Analysis of a synthetic, controlled case suggests that the accuracy of the regional attribution increases with increasing number of sites and record lengths, decreases with increasing regional heterogeneity, increases with increasing difference of the scaling fingerprints, and decreases with an increase of their prior uncertainty. The applicability of the framework is illustrated for a case study set in Austria, where positive flood trends have been observed at many sites in the past decades. The individual scaling fingerprints related to the atmospheric, catchment, and river system processes are estimated from rainfall data and simple hydrological modeling. Although the distributions of the contributions are rather wide, the attribution identifies precipitation change as the main driver of flood change in the study region. Overall, it is suggested that the extension from local attribution to a regional framework, including multiple drivers and explicit estimation of uncertainty, could constitute a similar shift in flood change attribution as the extension from local to regional flood frequency analysis.

## Key Points

A new framework for consistently attributing flood changes in a region
Spatial scaling of flood changes allows attribution of multiple drivers
Bayesian framing allows estimation of the attribution uncertainties

## 1 Introduction

Numerous major floods around the world have raised the concern that river flooding is becoming more frequent and severe [e.g., *Kundzewicz et al*., 2013; *Hall et al*., 2014; *Blöschl et al*., 2015; *Stevens et al*., 2016]. While there is consensus that in many parts of the world, the flood damage has increased due to an increase in the assets in the floodplains [e.g., *Jonkman*, 2005; *Di Baldassarre et al*., 2010; *Mechler and Bouwer*, 2014; *Ceola et al*., 2015], the actual changes in the flood hazard (associated with changed flood discharges) are less clear. A number of studies have examined whether significant river flood changes are detectable from long flood records [*Kundzewicz*, 2012; *Hall et al*., 2014; *Mediero et al*., 2015]. Regional patterns of changes do appear, but it is difficult to identify the reasons of such changes. *Merz et al*. [2012b] therefore called for a concerted effort for attributing trends in flood time series. Knowledge of the drivers of past flood changes will greatly enhance the capability of anticipating future flood changes [*Hall et al*., 2014].

River floods are affected by numerous processes and any changes in such processes may result in changes in the flood discharges. *Merz et al*. [2012a, 2012b] defined three groups of potential drivers related to the atmosphere, catchments, and the river system [see *Merz et al*., 2012b, Table 1].

*Atmosphere*. Any change in convective rainfall, synoptic rainfall, and snowmelt will induce changes in flood magnitudes. Additionally, antecedent soil moisture as controlled by antecedent precipitation and evaporation will play a role.

*Catchments*. Land use has changed considerably in many areas around the world, for example, due to deforestation and urbanization. Agricultural practices have dramatically affected water flow paths, e.g., through reducing infiltration by soil compaction.

*River systems*. Rivers have been manipulated for centuries by river training, removal of inundation areas, and construction of weirs which will all affect flood wave propagation and retention, and therefore the peak discharges. *Hall et al*. [2014] suggested how these drivers may affect river flood changes for different catchment scales and event magnitudes.

Which of these drivers are important for explaining observed river flood changes depends on the local situation. *Merz et al*. [2012b] discussed the state of the art in attributing observed flood changes to their drivers. They concluded that most studies focused on the detection problem, by performing statistical analyses of flood time series, and based their attribution statement on qualitative reasoning or even speculation. Hence, the observed flood change was explained by qualitatively linking it to changes in potential drivers, such as atmospheric circulation patterns [*Petrow et al*., 2009; *Bormann et al*., 2011], precipitation [*Robson et al*., 1998; *Mudelsee et al*., 2003], river training [*Villarini et al*., 2011], construction of reservoirs [*Bormann et al*., 2011], or changes in land use and agricultural management [*van der Ploeg and Schweigert*, 2001; *Pinter et al*., 2006; *Bormann et al*., 2011].

There are a number of studies that link changes in floods to their potential drivers via statistical approaches. Often, mean flood characteristics are correlated to meteorological variables [e.g., *Cunderlik and Burn*, 2004; *Pinter et al*., 2006; *Novotny and Stefan*, 2007] or to land use changes in paired catchments [see, e.g., *Alila et al*., 2009, and references therein]. Alternatively, nonstationary extreme value statistics are used for the extreme characteristics of floods [e.g., *North*, 1980; *Khaliq et al*., 2006; *Serinaldi and Kilsby*, 2015; *Šraj et al*., 2016]. The underlying idea is to allow the parameters of the flood frequency distribution to change in time as a function of a covariate and test whether this allows a better fit to the data. Even though the main objective is flood frequency estimation, the selection of the best covariate can be considered as attribution. For example, *Delgado et al*. [2012, 2014] attributed the trends in flood peaks for gauges along the Mekong River by using a GEV distribution whose scale parameter is a function of the variance of an atmospheric circulation index representing the strength of the monsoon system. Similarly, *Micevski et al*. [2006] used this approach to attribute flood changes in Australia to the Interdecadal Pacific Oscillation (IPO).

Besides these data‐based, statistical approaches, simulation models have been used to attribute observed changes to their potential drivers. The basic idea is to compare two scenarios, with and without the effect of the driver, and assess their consistency with the observed flood changes. *Renard et al*. [2008] attributed in this way flood trends in four catchments in France to changes in rainfall, while *Hamlet and Lettenmaier* [2007] found that flood changes in the western U.S. could be attributed to temperature changes in the twentieth century. *Hundecha and Merz* [2012] extended this approach by introducing the effect of year to year natural climate variability in the attribution, which allowed them to compare probability distributions of simulated flood changes, due to changes in meteorological variables, to the observed flood changes. The simulation‐based attribution approach has also been used for evaluating the effects of nonclimatic drivers, such as effects of afforestation and deforestation [*Andréassian et al*., 2003] or effects of river training [*Vorogushyn and Merz*, 2013].

While the attribution studies mentioned above have focused on a single driver, a number of multidriver studies have been published recently. Statistical approaches have been developed based on assessing the significance of more than one covariate in explaining the temporal variability of flood frequency model parameters through linear and nonlinear regression models. For example, *Villarini and Strong* [2014] and *Prosdocimi et al*. [2015] considered both precipitation and a land use indicator as covariates, in a US and UK context, respectively. While *Villarini and Strong* [2014] attributes flood changes to rainfall variability changes, in *Prosdocimi et al*. [2015] the urbanization effect has a dominant role. *Silva et al*. [2015] considered both ENSO and the construction of dams over the Itajaí‐açu River in Brazil. They provide evidence that upstream dams play a significant, if small, role in reducing flood hazard, while the increase in ENSO amplitude in the last decades has brought about a much stronger increase of flood magnitudes. Simulation‐based approaches coupled with trend detection tests have instead been used, for example, by *Harrigan et al*. [2014] and *Jia et al*. [2012], who explored a set of potential drivers of change in annual mean and high flows in one catchment in Ireland and China, respectively. In both studies, local human activity (land use change, field drainage, and artificial water use) was identified as principal responsible for the changes in runoff.

Most of the studies mentioned above have analyzed catchments individually, but there are a number of studies that have attempted to use regional information for attributing flood changes. Typically, these studies use a large set of streamflow time series and investigate whether regional patterns in the flood changes can be found. One approach first clusters the streamflow gauges and then derives flood changes for the clusters [e.g., *Mediero et al*., 2015]. Another approach investigates whether there are spatial patterns in the flood trends of many stations that can be interpreted in terms of attribution statements (e.g., seasonal coherence and consistency with large‐scale climatic driver in *Petrow and Merz* [2009]).

Also regional nonstationary flood frequency analyses have been developed that analyze many sites simultaneously and, typically, climate indices [*Kiem et al*., 2003; *Sun et al*., 2014, 2015] or directly atmospheric or oceanic fields [*Renard and Lall*, 2014] as covariates. These analyses include an evaluation of the uncertainty of the flood frequency model parameters and quantiles, usually by Bayesian statistics. They typically compare estimated stationary and nonstationary flood frequency curves with uncertainty through model selection criteria [e.g., *Renard et al*., 2006a, 2006bb; *Ouarda and El Adlouni*, 2011]. For the selected models, they then assess the credibility of the relationships between covariates and model parameters [e.g., *Sun et al*., 2014; *Silva et al*., 2015]. However, these analyses are more an exception than a rule among attribution approaches in the flood change literature. In fact, in most cases any quantitative confidence statement on the contribution of drivers is provided [*Merz et al*., 2012b].

The aim of this paper is to propose a new method of flood change attribution that accounts for multiple drivers, takes a regional perspective, and provides a confidence statement about the strength of the attribution. Specifically, the proposed framework exploits the differences of flood changes between catchments of different sizes. Section 2 describes the framework and the specific models adopted here. Section 3 explores the potential of the framework for a synthetic study. Section 4 illustrates the applicability of the framework for a real‐world case study, and section 5 discusses the main outcomes.

## 2 Attribution Framework

### 2.1 The Concept of Regional Flood Attribution Based on Scaling Fingerprints

There are numerous research questions in the geosciences where one is interested in identifying the processes or sources that contribute to an observed signal. Fingerprinting is a method that is specifically geared toward attributing these sources. For example, “…sediment source fingerprinting applied to fluvial systems aims to provide information on the source of the sediment transported by a river. It involves collecting a sample of the sediment transported by a river and comparing its physical or geochemical properties with those of potential sources within its catchment area. By matching the fingerprint of the sediment to those of the potential sources, it is possible to obtain information on the likely source or provenance of the sediment or, perhaps more likely, the relative importance of several different sources” [*Walling*, 2013, p. 1658]. In a similar vein, chemical [*Elsenbeer et al*., 1995] and bacterial [*Savio et al*., 2015] fingerprinting has been used to identify the sources of water, sea level fingerprinting to identify the mass sources of sea level rise [*Riva et al*., 2010], sediment fingerprinting for attributing nonpoint source pollution [*Davis and Fox*, 2009], and climate fingerprinting to identify anthropogenic climate change [*Hegerl et al*., 1996, 1997; *Levine and Berliner*, 1999; *Hidalgo et al*., 2009]. In hydrology, fingerprinting has been used to attribute changes of water resources to potential drivers [*Jia et al*., 2012] and to study watershed erosion processes [*Fox and Papanicolaou*, 2008].

In fingerprinting, the problem is framed as an inverse problem with two main assumptions: (a) the resulting signal is a mixture of component signals and (b) the patterns of the component signals (i.e., the fingerprints) are known to some degree. The accuracy of the estimated source contributions will depend on whether the inverse system is well or ill posed (i.e., how sensitive the resulting signal is to the component signals), the uncertainties involved, applicability of the mixing assumptions, and how realistic the fingerprints are.

Attributing changes in the river flood regime to their drivers is a typical fingerprinting problem. The observed signal is the change in the time series of the river flood discharges. The component processes are the changes in the drivers.

In our framework the fingerprints are conceptualized as the scaling characteristics with area of the driver components of flood change. While scaling of flood peak flows statistics with catchment area is known since long time in hydrology [e.g., *Benson*, 1963; *Alexander*, 1972; *Blöschl and Sivapalan*, 1995; *Robinson and Sivapalan*, 1997a, 1997bb; *Gupta et al*., 2007] and studies exist that address aspects of spatial scaling, nonstationarity, and uncertainty analysis [e.g., *Lima and Lall*, 2010], scaling fingerprints are used here for the first time. They represent our knowledge/assumption on how different drivers affect flood changes in catchments of different sizes. The scaling fingerprints are therefore associated with many catchments in a region. They can be used for regional flood change attribution, rather than for attribution in individual catchments. One would expect different scaling characteristics for the different drivers as suggested by *Blöschl et al*. [2007]. Some examples follow:

*Atmosphere* (*A*). Increases in local, convective precipitation are more relevant in small catchments which may lead to a decrease of flood changes with catchment area (smaller flood changes in larger catchments). Increases in regional precipitation due to changed atmospheric circulation patterns are more relevant in large catchments which will lead to an increase of flood changes with catchment area. Overall, these two effects may balance leading to small differences of flood change for catchments of different sizes.

*Catchment* (*C*). Decreasing infiltration capacity of the soil due to agricultural soil compaction and urbanization may increase floods in small catchments. Due to agricultural soil compaction and urbanization in the last decades, one would therefore expect an increase in flood discharges in these catchments. As the catchment size increases, the variety of land uses becomes larger (including nonagricultural and nonurban), so the effect will be less pronounced.

*River system* (*R*). Decreasing flood retention due to river training and loss of flood retention volumes in the floodplains may be a particularly relevant for low land rivers where settlement pressure is largest. There are usually large catchment areas associated with these rivers. Because of this, the effect of river works may be typically assumed to increase with catchment area.

In this paper we adopt specific models for the scaling characteristics (section 2.2) to illustrate the framework. Alternative scaling models could be used in a similar way. Similarly to regional flood frequency analysis [see, e.g., *Dalrymple*, 1960; *Burn*, 1988, 1990; *Hall and Minns*, 1999], the assumption of homogeneity within the region is very important. However, here the homogeneity does not only extend to the flood peak distributions and their response to controls [e.g., *Salinas et al*., 2014a, 2014b], but also in their response to temporal changes in the controls. In a homogeneous region, the drivers of flood change (e.g., atmospheric, catchment, and river system drivers) are assumed to change in the same way and to result in flood changes which are related to catchment size (see section 2.2 for a quantitative definition).

Attribution methods such as the one presented here involve a number of sources of uncertainty. These may be due to the limited flood record lengths (i.e., sample sizes), spatial heterogeneity within the region, uncertainties of the fingerprints, and uncertainties of the mixing model. The proposed approach is therefore framed in probabilistic, Bayesian terms (see, e.g., *Hasselmann* [1998], *Berliner et al*. [2000], *Lee et al*. [2005], and *Annan* [2010] for alternative Bayesian attribution approaches). The parameters of the fingerprint models are assumed to be random variables for which prior distributions are given. A Monte Carlo Markov Chain (MCMC) method is then used to update the distributions. The results are distributions of the contributions of the drivers to flood changes in the region. The narrower these distributions are, the more reliable is the attribution.

### 2.2 Model Assumptions

In this paper we aim at attributing the “regional expectation” of the temporal change of floods to the atmospheric, catchment, and river system drivers. More specifically, we consider changes in 〈ln⁡Q〉 (where 〈〉 indicates the expected value), where the random variable *Q* represents the (e.g., maximum annual) flood peak discharge, which varies in time and space (across different catchments). Notice that changes in the mean of the logarithm are close to percentage changes in the mean of the original variable (i.e., the correspondence would be exact for zero variance of the variable). In line with the fingerprinting concept, we assume that the flood change is a mixture of three components. In this paper, we adopt the linear mixing model

(1) $$\frac{d〈\ln Q〉}{dt}=\frac{d〈\ln A〉}{dt}+\frac{d〈\ln C〉}{dt}+\frac{d〈\ln R〉}{dt},$$

where d〈ln⁡A〉/dt, d〈ln⁡C〉/dt and d〈ln⁡R〉/dt represent the three components, i.e., the temporal change in the expectation of the log flood peaks if only one driver is present. Therefore, the random variables *A*, *C*, and *R* represent the flood peak discharges if only the atmospheric, catchment, or river processes were changing. We make here the additional assumption that the terms in equation (1) are constant in time and therefore correspond to linear trends of (the mean of) ln⁡Q, ln⁡A, ln⁡C, and ln⁡R (this assumption is not strictly necessary, since other types of change, including nonlinear trends or step‐changes, can be modeled analogously as long as these changes can be described by one coefficient, e.g., in this case the slope of the linear trend). Note that alternative mixing models could be used. In this paper, for clarity we focus on positive trends, i.e., regions where floods have increased in time, and our aim is to attribute this increase to the underlying causes. Of course, the fingerprint model can be extended for cases where negative or both positive and negative trends are of interest.

Given equation (1), the relative contribution of the atmosphere, catchment, and river components to flood peak changes is quantified by the coefficients

(2) $$\alpha =\frac{d〈\ln A〉/dt}{d〈\ln Q〉/dt},\chi =\frac{d〈\ln C〉/dt}{d〈\ln Q〉/dt},\rho =\frac{d〈\ln R〉/dt}{d〈\ln Q〉/dt},$$

whose estimation is the final aim of the multidriver attribution.

In this paper, the flood change components are related to catchment areas *S* through scaling relationships. Here we assume that

(3) $$\frac{d〈\ln A〉}{dt}=a_{A}S^{b_{A}},\frac{d〈\ln C〉}{dt}=a_{C}S^{b_{C}}\text{ and }\frac{d〈\ln R〉}{dt}=a_{R}S^{b_{R}},$$

where the location coefficients *a_A_*, *a_C_*, and *a_R_* represent the flood peak changes (i.e., trends in time) due to one driver for a catchment of unit area, and the scaling exponents *b_A_*, *b_C_*, and *b_R_* represent how the flood trends due to one driver change with catchment area. The exponents *b_A_*, *b_C_*, and *b_R_* are the scaling fingerprints whose knowledge allows the attribution of flood trends to the three drivers.

**Figure 1:**
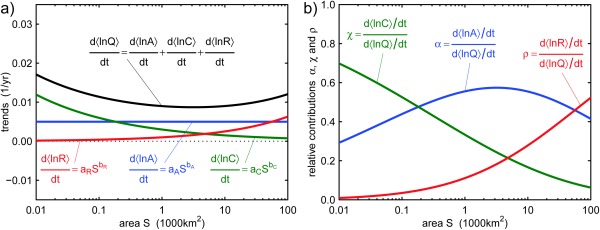
Schematic of a hypothetical mixing model and scaling behavior of flood trends (equations (1) and (3)): (a) flood trends ( d〈ln⁡Q〉/dt) and trend components ( d〈ln⁡A〉/dt, d〈ln⁡C〉/dt and d〈ln⁡R〉/dt) as a function of catchment area *S*; (b) relative contributions of the atmosphere (*α*), catchment (*χ*), and river (*ρ*) components of flood peak trends as a function of catchment area *S*. The colorcode for this and all following figures: blue = atmosphere, green = catchment, and red = river.

Since we focus on driver contributions to positive flood peak changes, *a_A_*, *a_C_*, and *a_R_* are assumed to be greater than zero (drivers acting in opposite directions could be included in the model, but the problem would become less well posed). For the same reason, also *α*, *χ*, and *ρ* are positive, and α+χ+ρ=1. As an illustration, Figure 1a shows hypothetical components and their combination into the scaling behavior of flood changes. Throughout the paper the units of *S* are 1000 km^2^. The values of the parameters in Figure 1 are *a_A_* = 0.005 ${\text{km}}^{-2b_{A}}$ yr^−1^, *a_C_* = 0.003 ${\text{km}}^{-2b_{C}}$ yr^−1^, *a_R_* = 0.001 ${\text{km}}^{-2b_{R}}$ yr^−1^, *b_A_* = 0, *b_C_* = −0.3, and *b_R_* = 0.4. In this hypothetical region, the climate component of flood changes (blue line in Figure 1a) does not vary with catchment area, the catchment component (green line) is strong for small catchments and gets weaker with increasing catchment size, and the river component (red line) is small for small catchments and gets stronger with increasing catchment size. The shape of the three lines is related to the scaling fingerprints *b_A_*, *b_C_*, and *b_R_*. The relative contribution of the three drivers, as a function of catchment area, is shown in Figure 1b. In small catchments, catchment and atmospheric processes are the most important drivers of flood change. In intermediate catchments atmospheric processes are dominant, and in large catchments atmospheric and river system processes.

Given a region with data on flood trends at many sites, the next step is to estimate the parameters of the model of equations (1) and (3) that better explain the regional pattern of the data (see section 2.3). In this paper we make assumptions on the scaling fingerprints *b_A_*, *b_C_*, and *b_R_*. We have chosen not to make assumptions on the location coefficients *a_A_*, *a_C_*, and *a_R_* in order to demonstrate the value of the information on the scaling exponents alone (i.e., the fingerprints), which we hypothesize to be more universal than the location coefficients, in a similar fashion as the shape parameter of the flood frequency curve in regional flood frequency analysis is more universal than the other parameters [see, e.g., *Fiorentino et al*., 1987; *Martins and Stedinger*, 2000].

Similarly to regional flood frequency analysis, we make here the assumption of regional homogeneity, which implies that the scaling relationships in equation (3) apply to every catchment in the region. Therefore, the scatter that we may see in the data is considered just sampling variability due to the limited record length. This is analogous to the usual assumption of the index‐flood method [*Dalrymple*, 1960; *Hosking and Wallis*, 1997], i.e., that all moments but the mean (or median) of the flood frequency distributions in the region are the same.

### 2.3 Estimation Method

**Figure 2:**
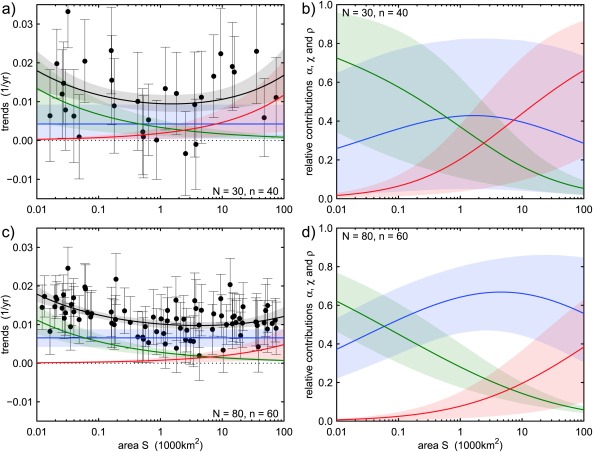
Bayesian flood trend attribution (estimation of *a_A_*, *a_C_*, and *a_R_*, and therefore *α*, *χ*, and *ρ*) using the MCMC model when the scaling fingerprints *b_A_*, *b_C_*, and *b_R_* are known. Estimation for two hypothetical (synthetic) regions: (a, b) homogeneous region with *N* = 30 sites with *n* = 40 years of flood data each; (c, d) homogeneous region with *N* = 80 sites with *n* = 60 years of flood data each. Mean (dark line) and 90% credible bounds (light transparent areas) for regional expected flood trends and trend components (a, c) and their relative contributions to flood trends (b, d) as a function of catchment area *S*. The full circles in Figures 2a and 2c represent the local observed trends and the vertical bars show their 90% confidence intervals. As in Figure 1, the colorcode: blue = atmosphere, green = catchment, and red = river.

In Figures 2a and 2c, two hypothetical regions are presented, whose number of sites *N* and record length *n* differ. Flood data *Q* are generated from lognormal distributions with parameters consistent with the scaling behavior of flood changes of Figure 1 (i.e., log‐means increasing with time and log‐standard deviation constant and equal to 0.5), and the local linear trends d〈ln⁡Q〉/dt are estimated by fitting a simple linear regression with the least squares method [*Kottegoda and Rosso*, 1997, pp. 341–357]. We indicate hereafter with $\overset{⁁}{d{〈\ln Q〉}_{i}/dt}$ the estimated flood trend and with ${\hat{\sigma }}_{d{〈\ln Q〉}_{i}/dt}$ its estimated standard deviation at site *i*, with i=1,…,N. The full circles in Figures 2a and 2c represent the locally estimated trends and the vertical bars show their 90% confidence intervals.

Given a region with observed positive flood peak trends for *N* sites and the attribution mixing model used here (combining equations (1) and (3)), the posterior distribution of the parameters follows from the Bayes' equation

(4) $$p(\theta |D)=\frac{\ell (D|\theta )\pi (\theta )}{\int_{\Omega }\ell (D|\theta )\pi (\theta )d\theta }\propto \ell (D|\theta )\pi (\theta ),$$

where $\theta =(a_{A},a_{C},a_{R},b_{A},b_{C},b_{R})$ are the parameters of the mixing model and D are the observations (i.e., the locally estimated flood trends, their estimated standard deviation and catchment areas). The likelihood function is

(5) $$\ell (D|\theta )=\prod_{i=1}^{N}f_{N}\left(\hat{\frac{d{〈\ln Q〉}_{i}}{dt}};a_{A}S_{i}^{b_{A}}+a_{C}S_{i}^{b_{C}}+a_{R}S_{i}^{b_{R}},{\hat{\sigma }}_{d{〈\ln Q〉}_{i}/dt}\right),$$

where $f_{N}(x;\mu ,\sigma )$ is the normal density function with mean *μ* and standard deviation *σ*. The regional expectation of flood trends is, for site *i*, $a_{A}S_{i}^{b_{A}}+a_{C}S_{i}^{b_{C}}+a_{R}S_{i}^{b_{R}}$, which contains the scaling with area of the flood change components. With π(θ), in equation (4) we indicate the prior distribution of the parameters. The idea is that if the scaling fingerprints *b_A_*, *b_C_*, and *b_R_* are fully or partially known, and if their values differ significantly, fitting the attribution model in equations (1) and (3) becomes a well‐posed problem and can be solved. An informative prior normal distribution for the scaling fingerprints *b_A_*, *b_C_*, and *b_R_* is used. In contrast, the parameters *a_A_*, *a_C_*, and *a_R_* are assumed to be unknown, with an improper uniform prior distribution over the entire positive real line. In this paper we assume independence between flood trends at different sites, but spatial correlation could be accounted for by replacing ${\hat{\sigma }}_{d{〈\ln Q〉}_{i}/dt}^{2}$ by a covariance matrix. Also, we use independent prior distributions for the parameters, including the scaling fingerprints. Note that equations (4) and (5) do not account for uncertainty in the linear mixing model, whose structure is assumed correct, apart from the uncertainty in its parameters.

Since the integral in the denominator of equation (4) cannot be processed in closed form, we use here the Hamiltonian Monte Carlo sampling procedure [*Duane et al*., 1987; *Neal*, 1994, 2011; *Stan Development Team*, 2015a]. The code used in this work is described in Appendix A. Figure 2 shows the result of applying the Bayesian estimation procedure to the two hypothetical regions with different data availability. The units of *S* have been chosen as 1000 km^2^ because this is about the mean logarithmic area in the regions of interest here and reduces the correlation between the parameters in equation (3), thus facilitating the convergence of their estimates [*Lima and Lall*, 2010]. Figure 2 shows mean and 90% credible bounds of the posterior distributions of the regional expectation of flood peak changes, their components (Figures 2a and 2c), and their relative contributions *α*, *χ*, and *ρ* (Figures 2b and 2d) for the two hypothetical regions, when the scaling fingerprints *b_A_*, *b_C_*, and *b_R_* are assumed to be known exactly and correspond to their “real” values, i.e., those used to generate the flood peak samples. Essentially, in Figure 2 only the values of *a_A_*, *a_C_*, and *a_R_* are fitted to the data. It can be seen that for the region with more sites and longer record lengths (Figures 2c and 2d) the attribution is much more precise (i.e., narrower confidence bounds) and accurate (i.e., closer to reality as shown in Figure 1) than for the region with fewer sites and shorter record lengths (Figures 2a and 2b). It is therefore of interest to assess the sensitivity of the attribution method to the available information, which is done in the following section.

## 3 Synthetic Case: Sensitivities and Value of the New Framework

We repeat here the exercise of flood change attribution shown in Figure 2 many times to investigate the sensitivity of the framework to the number of sites in the region, to the record lengths, and to regional heterogeneity (section 3.1). Also, we investigate the sensitivity of the framework to the strength of the scaling of the flood change components with catchment area (i.e., strength of the fingerprints) and to the degree of uncertainty of our prior information on that scaling (section 3.2).

The procedure is as follows:

1. Generate flood peak time series for a large number of regions (here 2000) with one characteristic of interest (e.g., the number of sites in the region) varying over a predefined range. The flood peak data *Q* are generated from lognormal distributions with parameters consistent with the scaling behavior of the flood changes of equations (1) and (3) and log‐standard deviation equal to 0.5, as in Figure 2.
2. For each site in each region, estimate the temporal trend of the mean‐log of the flood peaks $\hat{d{〈\ln Q〉}_{i}/dt}$ and its standard deviation ${\hat{\sigma }}_{d{〈\ln Q〉}_{i}/dt}$ using the least squares method.
3. For each region, perform the flood change attribution as described in section 2.3, i.e., estimate *a_A_*, *a_C_*, *a_R_*, *b_A_*, *b_C_*, and *b_R_*, using a priori information on the scaling fingerprints *b_A_*, *b_C_*, and *b_R_* with the MCMC procedure. Here 1000 realizations of the posterior distribution of the parameters are sampled with the MCMC procedure, which correspond to 1000 realizations of the posterior distribution of *α*, *χ*, and *ρ* (the convergence of the MCMC is diagnosed visually through multiple chain trace plots).
4. For each region, and for different values of catchment area (here for *S* = 0.001, 0.01, 0.1, 1, 10, 100, and 1000), calculate the mean and standard deviation of the posterior distribution of *α*, *χ*, and *ρ*.
5. Group the results according to the one characteristic of interest (here to 10 groups containing approximately 200 regions each) and, for each group, calculate the spatially averaged (for varying *S*) bias and standard deviation of the *α*, *χ*, and *ρ* estimates.
6. Plot the average bias and standard deviation, along with the average standard deviation of the posterior distribution of *α*, *χ*, and *ρ* obtained with the MCMC procedure.

The performance of the method can then be visually assessed by checking how close is the bias to 0 and how well the standard deviation of the posterior distribution of *α*, *χ*, and *ρ* (i.e., the estimated uncertainty) fits the mean standard deviation of estimation (i.e., the expected error variability). This latter is done to check whether the uncertainty estimated by the Bayesian approach is comparable to the uncertainty one would expect, in a frequentist sense, from sampling variability. For zero bias, estimated uncertainty lower than expected error variability (respectively indicated as “est. sd” and “exp. sd” in the following figures) would indicate an overly confident fitting procedure. In the opposite case, for zero bias, estimated uncertainty greater than expected error variability would indicate an overestimation of the uncertainty by the method.

### 3.1 Pooling, Record Lengths, and Regional Homogeneity

**Figure 3:**
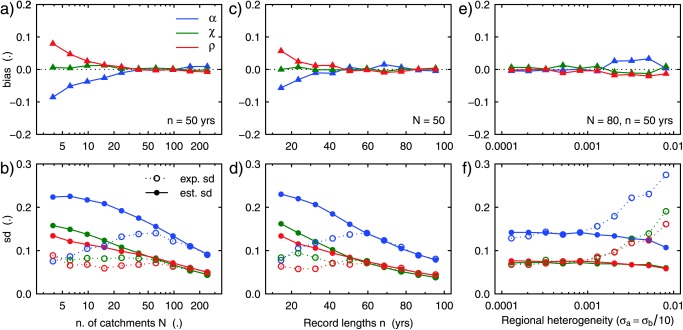
Bias (top row) and standard deviation (bottom row) of the estimated relative contributions *α*, *χ*, and *ρ* of atmospheric, catchment, and river drivers to flood trends for (a, b) varying number *N* of sites in the region; (c, d) varying record length *n*; and (e, f) varying regional heterogeneity. The performance of the method can be visually assessed by checking how close is the bias to 0 and how well the estimated standard deviation (est. sd) fits the expected standard deviation (exp. sd). *σ_a_* and *σ_b_*, in Figures 3e and 3f, are the standard deviations for the lognormal and normal distributions used to generate noise in the local values of the *a* and *b* parameters.

To illustrate the sensitivity of the method to the number of sites in the region, homogeneous regions have been generated from the model in Figure 1 with a different number of sites (from 3 to 300) and record lengths of 50 years. The regions are homogeneous in that the scaling relationships in equation (3), with the same set of parameters, apply to every catchment in the region. Notice that for the regional attribution of flood trends to three drivers, data of at least three sites are needed. In this section, we assume to know exactly the scaling fingerprints *b_A_*, *b_C_*, and *b_R_* (i.e., prior distributions are Dirac‐deltas, and the prior information is unbiased). In Figures 3a and 3b the bias, the standard deviation of estimation (exp. sd), and the mean standard deviation of the posterior distribution (est. sd) for the estimation of *α*, *χ*, and *ρ* are shown in relation to the number of sites in the region. For a small number of sites, the relative contribution of the atmospheric driver is underestimated, especially for large catchments (not shown here) where the river works contribution is overestimated (similarly to Figure 2b as compared to Figure 1b). The standard deviation of the error is low since the estimated values of *α*, *χ*, and *ρ* tend to be concentrated around their a priori values. With a record length of 50 years, homogeneous regions of at least 40 sites are required to obtain an unbiased estimation of *α*, *χ*, and *ρ*, and at least 100 sites are required for an unbiased estimation of their variability (measured by the expected standard deviation of estimation).

Figures 3c and 3d are similar to Figures 3a and 3b but show the sensitivity of the attribution methodology to the record length (from 10 to 100 years) for regions with 50 sites. With record lengths of at least 50 years the method provides unbiased estimates of *α*, *χ*, and *ρ*, and with 60 years the estimation variability is correctly captured.

Figures 3e and 3f show the sensitivity of the method to the regional heterogeneity, which is defined as the spatial variability of the scaling laws of the fingerprints within the region. The heterogeneity is generated in the data by adding noise to the values of *a_A_*, *a_C_*, *a_R_*, *b_A_*, *b_C_*, and *b_R_*. The noise for the location coefficients *a* is generated by a lognormal distribution with mean equal to *a_A_*, *a_C_*, and *a_R_* and standard deviation *σ_a_*. The noise for the scaling fingerprints *b* is generated by a normal distribution with mean equal to *b_A_*, *b_C_*, and *b_R_* and standard deviation $\sigma_{b}=10\sigma_{a}$. Figure 3e shows that the bias is not strongly affected by the heterogeneity. This is because the average regional values of the scaling fingerprints are correctly guessed (a priori). However, the expected standard deviations of *α*, *χ*, and *ρ* are increasingly underestimated (Figure 3f), once the heterogeneity *σ_a_* exceeds 0.001. Notice that this value corresponds to 20% of *a_A_*, 30% of *a_C_*, and 100% of *a_R_*. This suggests that the estimation is not very sensitive to the simulated regional heterogeneity.

### 3.2 Scaling Fingerprints

**Figure 4:**
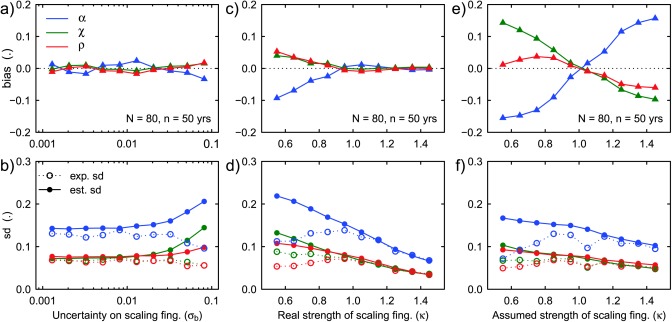
Bias (top row) and standard deviation (bottom row) of the estimated relative contributions *α*, *χ*, and *ρ* of atmospheric, catchment, and river drivers to flood trends for (a, b) varying uncertainty of the prior information on the fingerprints *b_A_*, *b_C_*, and *b_R_*; (c, d) varying difference between the “real” *b_A_*, *b_C_*, and *b_R_*; and (e, f) varying bias in the prior information on the fingerprints *b_A_*, *b_C_*, and *b_R_*. In Figures 4a and 4b *σ_b_* is the standard deviation of the prior normal distribution of the *b* parameters. In Figures 4c and 4d the regions are generated from equations (1) and (3), with parameters *b_A_* = 0, *b_C_* = −0.3 ·κ, and *b_R_* = 0.4 ·κ, and the prior information on the scaling fingerprints is correct: *b_A_* = 0, *b_C_* = −0.3 ·κ, and *b_R_* = 0.4 ·κ. In Figures 4e and 4f the regions are generated with parameters *b_A_* = 0, *b_C_* = −0.3, and *b_R_* = 0.4, and the prior information on the scaling fingerprints is biased: *b_A_* = 0, *b_C_* = −0.3 ·κ, and *b_R_* = 0.4 ·κ.

Figures 4a and 4b show the effect of the degree of uncertainty in our knowledge of the scaling fingerprints *b_A_*, *b_C_*, and *b_R_*. In contrast to Figures 3e and 3f, the regions are homogeneous and correspond to the model shown in Figure 1, but the a priori information on *b_A_*, *b_C_*, and *b_R_* is modeled as a normal distribution with mean equal to the correct values of *b_A_*, *b_C_*, and *b_R_* and standard deviation *σ_b_*. The results are comparable to those in Figures 3e and 3f with the difference that here the estimated standard deviation tends to become larger than the expected standard deviation of estimation when *σ_b_* grows larger.

Figures 4c and 4d address the issue of how well posed the attribution problem is by examining the effect of the difference between the scaling fingerprints *b_C_* and *b_R_*. Here *b_A_* = 0, *b_C_* = −0.3 ·κ, and *b_R_* = 0.4 ·κ, where *κ* represents the strength of the scaling of the catchment and river flood change components with area relative to each other. When *κ* = 1, the samples are generated from the model shown in Figure 1. Larger *κ*'s represent regions where the difference between the scaling of catchment and river effect is steeper, smaller *κ*'s where it is smaller. As *κ* approaches 0 the problem becomes more and more ill‐posed and the contributions can no longer be identified. In fact, the attribution procedure proposed here hinges on the assumption that the scaling effects on floods differ significantly between the drivers (i.e., that the scaling fingerprints *b_A_*, *b_C_*, and *b_R_* are distinct). If the strength of the fingerprints is only half of that assumed in the standard case (e.g., *κ* = 0.5), biases increase considerably but also the estimated standard deviations, thus indicating that the attribution is not feasible. A stronger contrast of the fingerprints (e.g., *κ* = 1.5) increases instead the identifiability with close to perfect estimation of *α*, *χ*, and *ρ*.

Figures 4e and 4f show the effect of assuming different scaling fingerprints *b_C_* = −0.3 ·κ and *b_R_* = 0.4 ·κ, while, in reality, they are those in Figure 1 (i.e., *κ* = 1). *κ* measures here the bias in the prior information on the scaling fingerprints. The stronger this bias, the stronger the bias of estimation of *α*, *χ*, and *ρ*. This is to be expected since the method makes use of the prior information on the scaling fingerprints. Interestingly, for large values of *κ*, the correspondence between the estimated standard deviation of the expected standard deviations of estimation becomes closer. This has probably to do with *α*, *χ*, and *ρ* being related, i.e., their sum is equal to 1, and bounded between 0 and 1.

## 4 Real Case Study

### 4.1 Study Area and Data

To illustrate the feasibility of the method, a real‐world case study is presented here. The study area is Upper Austria, where mostly positive flood trends have been detected in the last decades [*Blöschl et al*., 2011, 2012]. At this stage, the drivers of these trends are not clear but potential candidates are increases in rainfall, possibly associated with changes in atmospheric patterns [*Bárdossy and Caspary*, 1990; *Petrow et al*., 2009], land use changes as a result of intensification of agriculture with heavier machinery [*Nawaz et al*., 2013], and construction of levees along the rivers to protect the floodplains [*Blöschl et al*., 2013]. The new framework is used to identify the relative contribution of these drivers to the overall flood change in the region, based on the data at hand.

Flood peak data for 97 river gauges, with areas ranging from 10 to 79,500 km^2^ and records of at least 40 years after 1950, are used. A simple linear trend analysis, based on fitting a regression line by the least squares method, shows that most of the stations have significant positive trends in the log of the flood peaks (black points and confidence bounds in Figures 6a and 6c).

### 4.2 Estimation of Scaling Fingerprints

This section deals with the problem of getting “prior” information about the scaling fingerprints *b_A_*, *b_C_*, and *b_R_* associated with the atmospheric, catchment, and river effects on flood changes. This is done through simplified rainfall‐runoff modeling using observed rainfall data but without using the flood peak data, as the latter are used for attribution. To obtain prior information for each component, we introduce change separately in the three components. For the atmospheric fingerprint we drive a constant catchment model with observed precipitation (including potentially changing precipitation), whereas for the catchment and river components we use detrended precipitation to drive models that include changes in land use or in retention volume of floodplains, respectively.

#### 4.2.1 A, Atmosphere

**Figure 5:**
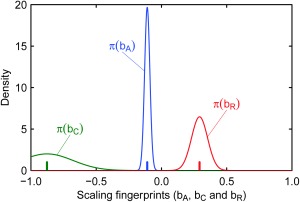
Scaling fingerprints *b_A_*, *b_C_*, and *b_R_* obtained through simplified rainfall‐runoff modeling (section 4.2) and to be used as prior information in the Bayesian regional attribution (section 4.3). For exact prior information on the scaling fingerprints (Figures 6a and 6b), the mean values represented by the spikes are used. For uncertain prior information on the scaling fingerprints (Figures 6c and 6d), normal distributions with mean and variance equal to those of the density functions shown here are used.

We calculate hourly catchment precipitation for all catchments in the study region from hourly and daily precipitation data of more than 900 rainfall stations in Austria and Bavaria, using the interpolation method of *Merz et al*. [2006, pp. 592–593]. We then run a number of moving windows of time length *τ* on the precipitation time series and calculate the precipitation sum for each window. This results in a filtered precipitation time series for each catchment and each *τ* value. We then assume that the duration of rainstorms triggering annual floods depends on the catchment size [*Viglione and Blöschl*, 2009] according to $\tau =a_{\tau }S^{b_{\tau }}$ with $a_{\tau }$ = 20 and $b_{\tau }$ = 0.3 [*Robinson and Sivapalan*, 1997a, equation (30)], where *τ* is in hours and *S* in 1000 km^2^. For a catchment of size *S*, we then select the maximum annual precipitation from the precipitation time series associated with the *τ* of this area (e.g., the maximum annual 24 h precipitation for a catchment of about 2000 km^2^). In a second step, we calculate (hypothetical) flood peaks *A* assuming a similar hydrograph shape for all events and the following runoff generation model:

(6) $$A(t)=b_{f}+\max\left[(i(t)-i_{c}),(h(t)-h_{c})/\tau ,0\right],$$

where *i_c_* and *h_c_* are (constant) parameters of the model and *b_f_* is a (constant) base flow. The first term in the square brackets represents the effect of infiltration excess, and the second term the effect of saturation excess. *i*(*t*) and *h*(*t*) are the maximum annual precipitation intensities and depths, respectively. For a given year i(t)=h(t)/τ, so that the quantities in the square brackets differ only due to the constant parameters *i_c_* and *h_c_*. By increasing the catchment size, *τ* increases and we expect increasing saturation excess and decreasing infiltration excess. We use here *i_c_* = 1.9 mm/h, *h_c_* = 10 mm, and *b_f_* = 0.05 mm/h. These parameters have been guided by the event analysis of *Merz et al*. [2006] and imply that the transition from infiltration excess (in small catchments) to saturation excess dominance (in larger catchments) occurs at a catchment scale of 14 km^2^. As a third step we estimate, for each catchment separately, the time trend of ln⁡A, i.e., $\hat{d〈\ln A〉/dt}$, and the uncertainty ${\hat{\sigma }}_{d〈\ln A〉/dt}$ of the trend, by fitting a linear regression model with the least squares method. As a fourth step we pool all the catchments in the region and estimate *a_A_* and *b_A_* (with uncertainty) using the MCMC procedure analogous to equations (4) and (5) but with the atmospheric component only. An improper uniform prior distribution over the entire positive real line is used for *a_A_*, and a flat normal prior distribution with mean 0 and standard deviation 100 is used for *b_A_*. The result of the fitting of *b_A_* (the atmospheric scaling fingerprint) to the data is shown in Figure 5, where the estimated mean is represented by the blue vertical spike and the distribution is represented by the blue line. The decreasing atmospheric effect on flood changes with catchment area, which appears here small but significant, could be related to the hypothesis that climate change has bigger effects in small catchments due to increasing convective precipitation, which is more relevant in these catchments. Note that we do not estimate the atmospheric fingerprint from the flood data, as they would include also the effects of catchment and river changes. The estimated mean and distributions in Figure 5 will then be used as prior information on the scaling fingerprints in the attribution exercise of section 4.3.

#### 4.2.2 C, Catchment

We assume here that the main effect of land use change on floods comes from a modified infiltration capacity of the soil as a result of urbanization and agricultural soil compaction. For floods in small catchments, short storms with high intensities tend to be relevant, so the controlling runoff generation mechanism is infiltration excess. In larger catchments the flood‐relevant storms have lower intensities, so saturation excess becomes more relevant.

We detrend the maximum annual catchment precipitation time series associated with *τ* (which is a function of area) from the atmosphere (*A*) analysis above. We call the detrended precipitation intensities and depths $i^{*}$ and $h^{*}$, respectively. In a second step, we calculate (hypothetical) flood peaks *C* (assuming a similar hydrograph shape for all events) using the runoff generation model

(7) $$C(t)=b_{f}+\max\left[(i^{*}(t)-i_{c}(t)),(h^{*}(t)-h_{c})/\tau ,0\right],$$

for each catchment and each year. In the case of catchment effects, $i_{c}(t)$ is no longer constant with time due to soil compaction and surface sealing. We use the same values for *h_c_* and *b_f_*, as in section 4.2.1, and assume that *i_c_* decreases by 10% per decade [*Strudley et al*., 2008] over the period of interest, from an initial value of 1.9 mm/h. As a third step we estimate, for each catchment separately, the time trends of ln⁡C and their uncertainties. As a fourth step we pool all the catchments in the region and estimate *a_C_* and *b_C_* (with uncertainty) as before. The result of the fitting of *b_C_* to the data is shown in Figure 5 by the green spike and line. The catchment effect on flood changes decreases significantly with catchment area (i.e., the scaling fingerprint *b_C_* is negative), but the degree with which it does so is highly uncertain.

#### 4.2.3 R, River

We assume that the main effect of rivers on flood changes is due to the loss of retention volumes in the floodplain. This effect is stronger in larger catchments because of the construction of levees in the populated flatlands. The effect is also stronger for large floods as inundation tends to occur beyond a threshold (bankfull discharge).

We start from the flood time series as in equation (7) but without a trend (i.e., *i_c_* constant in time), here called $C^{*}$. It is assumed that the higher the protection level $I_{r}(t)$, the higher the amplification of the flood peaks due to loss of retention volume and altered flood conveyance [*Di Baldassarre et al*., 2009; *Remo et al*., 2012; *Heine and Pinter*, 2012]. This effect increases with catchment area, i.e.,

(8) $$R(t)=C^{*}(t)+a_{s}\cdot I_{r}(t-1)\cdot S^{b_{s}}.$$

The parameter *a_s_* can be estimated from hydrodynamic simulations of floodplain retention and is assumed as *a_s_* = 0.16 here, meaning that, for a 1000 km^2^ catchment the exacerbation of flood peaks is of 16% of *I_r_*. The parameter *b_s_* can be estimated from comparisons of catchments of different sizes and is assumed as *b_s_* = 0.3 here, which implies a doubling exacerbation effect on floods with the increase of 1 order of magnitude of the catchment size. The initial value of *I_r_*, at time 0, is chosen as 0. From this we estimate the temporal evolution of the protection level at the river based on the reasoning that levees are usually built in direct response to a major flood [*Di Baldassarre et al*., 2013; *Viglione et al*., 2014; *Di Baldassarre et al*., 2015]. The protection level $I_{r}(t)$ is assumed to increase directly after a flood occurs that is larger than the existing protection level

(9) $$I_{r}(t)=\{\begin{array}{cc} R(t) & \text{if}\;R(t)>I_{r}(t-1)\\ I_{r}(t-1) & \text{otherwise} \end{array}.$$

Notice that equations (8) and (9) are coupled, as in the socio‐hydrological model of *Di Baldassarre et al*. [2013], and the evolution of *R* and *I_r_* is governed by a positive feedback [*Sivapalan et al*., 2012; *Di Baldassarre et al*., 2015; *Sivapalan and Blöschl*, 2015]. Similar to the other drivers, we estimate for each catchment the time trends of ln⁡R and their uncertainties. We then pool all the catchments in the region and estimate *a_R_* and *b_R_* (with uncertainty) as before. The result of the fitting of *b_R_* to the data is shown in Figure 5 as the red spike and line. Clearly, the river works effect on flood changes increases significantly with catchment area (i.e., the scaling fingerprint *b_R_* is positive).

### 4.3 Results of the Case Study

**Figure 6:**
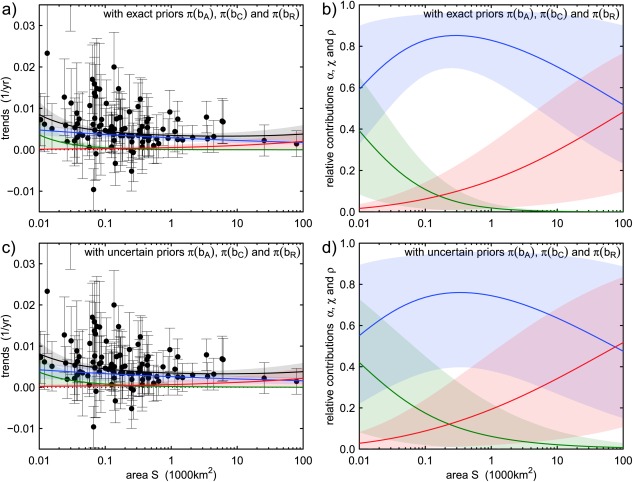
Bayesian flood trend attribution (estimation of *a_A_*, *a_C_*, and *a_R_*, and therefore *α*, *χ*, and *ρ*) of observed flood trends in Upper Austria. Estimation based on two assumptions: (a, b) exact prior information on the scaling fingerprints *b_A_*, *b_C_*, and *b_R_* (i.e., mean values in Figure 5); (c, d) uncertain prior information on the scaling fingerprints *b_A_*, *b_C_*, and *b_R_* (i.e., density functions in Figure 5). Mean (dark line) and 90% credible bounds (light transparent areas) for regional expected flood trends and trend components (a, c) and their relative contributions to flood trends (b, d) as a function of catchment area *S*. The full circles in Figures 6a and 6c represent the local observed trends and the vertical bars show their 90% confidence intervals. The colorcode: blue = atmosphere, green = catchment, and red = river.

Using the prior information on the three scaling fingerprints, we now apply the estimation method, as explained in section 2.3, to the observed flood trends in Upper Austria. We assume that the region is homogeneous, i.e., that a unique scaling with catchment area of the effects of the drivers on flood changes exists, which is captured by the model in equations (1) and (3) with parameters *a_A_*, *a_C_*, *a_R_*, *b_A_*, *b_C_*, and *b_R_*. Figure 6 shows the results of the regional attribution of flood changes (here 100,000 realizations of the posterior distribution of the model parameters are sampled with the MCMC procedure). In Figures 6a and 6b, the scaling fingerprints *b_A_*, *b_C_*, and *b_R_* are assumed to be known with certainty, with values set to the mean values of the distributions shown in Figure 5. In this case, the attribution method clearly separates the drivers indicating that overall, the main driver of change is climate, which is dominant at almost all scales, while catchment changes are as important as climate at small scales and river changes at large scales. Second (Figures 6c and 6d), the prior information on the scaling fingerprints *b_A_*, *b_C_*, and *b_R_* is used as obtained in section 4.2 and represented in Figure 5 by the density functions (i.e., with uncertainty). Due to the large uncertainty of the scaling fingerprints, the resulting attribution is associated with large uncertainty too. Similar conclusions can be made as for Figures 6a and 6b. The main driver of change is climate, which is dominant at almost all scales. This dominance is significant at intermediate catchment scales (i.e., 90% credible bands of *α* are above the others in Figure 6d). This is due to significant rainfall trends in the region with a scale dependence similar to that of flood trends.

## 5 Discussion and Conclusions

This paper goes beyond flood change attribution in several aspects. It proposes a rigorous framework for (1) multidriver attribution, i.e., for quantifying the contribution of different drivers of flood change, (2) regional attribution, i.e., transferring information from similar catchments to increase the signal‐to‐noise ratio, and for (3) providing a confidence statement about the strength of the attribution.

Although it is widely acknowledged that flood change may be caused by several drivers that act at the same time, multidriver attribution studies are rare [*Merz et al*., 2012b]. In this paper we have differentiated between three drivers representing the three compartments potentially responsible for river flood change: atmosphere, catchment, and river system. Each driver is understood as the aggregated effect of possibly several drivers of change within each compartment. It is important to note that our framework is not limited to this choice. The number of components and the components themselves can be varied. For instance, it could be used to separate the effects of urbanization from those of implementing retention storage in the catchment. Of course, the strength of the resulting attribution statements hinges on the disparity of the single components. Attribution will not be possible or will be very uncertain in case the single components leave a similar fingerprint on the flood change.

Most of the past studies on regional flood change attribution [see, e.g., *Mediero et al*., 2015; *Petrow and Merz*, 2009] have evaluated drivers in a qualitative way without providing quantitative estimates of the different contributions. In this paper we propose a framework that uses regional information of the change in a rigorous, quantitative way. The framework is based on the concept of scaling of flood change fingerprints with catchment area, i.e., it assumes that the drivers of flood changes produce effects that scale differently with catchment area.

Most current attribution approaches in the flood change literature do not provide confidence statements and simply explain observed changes without a quantitative measure of the uncertainty of the role of drivers [*Merz et al*., 2012b]. Our framework uses a Bayesian approach where scaling fingerprints of the different components are given as random variables. This results in a posteriori distribution for the percentage contribution of each component, representing the uncertainty of the attribution, under the assumptions of the methodology.

In this paper, we assume that the flood change behavior of the annual mean peak discharges is homogeneous within the region considered. We have mapped the space of feasible attribution conditions under which the method is able to identify the drivers of flood change to given accuracy and precision. Data pooling (increasing the number of sites and/or record length) increases the accuracy and precision of attribution to multiple drivers, i.e., reduces the bias and the width of the credibility bounds. The amount of data necessary to succeed in the attribution is comparable to what is available in densely gauged regions such as Europe. Attribution of flood changes requires, on one hand, a significantly strong signal‐to‐noise ratio in the regional flood trend data, so a larger number of catchments is advantageous. On the other hand, the attribution framework assumes that the spatial heterogeneity of the different drivers of flood change is small. This means, more specifically, that the percentage contribution of the drivers and their scaling with catchment area needs to be homogeneous among the pooled catchments. The simulations show that even if the region is mildly nonhomogeneous, the attribution is still close to unbiased and rather precise. This is important as regions cannot always be expected to be homogeneous with respect to their hydrological behavior. Homogeneity can be more easily expected for the atmosphere component where one can often assume that climate‐related flood changes are coherent across regions. This is less obvious for the catchment and river network components. For example, the catchment fingerprint in the case study is based on the simplified assumption that catchment effects can be represented by a steady decrease in soil infiltration capacity over the years which is the same in all catchments, yet this will not strictly be the case in real‐world regions.

The analyses in this paper demonstrate that information on the fingerprint scaling with catchment area alone may suffice for attributing the flood trends, even if no prior information on the magnitude of the trend components is available. By using information on the scaling fingerprints (and no information on the magnitude of the trend components), the real‐world case study gives plausible results. In this particular case study, precipitation change is the main driver of increasing flood trends in Upper Austria in the last decades which is consistent with the qualitative reasoning of *ZAMG and TU Wien* [2011]. For small catchments, land use change plays an important role (albeit smaller than climate) while for large catchments river works are important (again, smaller than climate effects). This change of process is plausible too, given the general understanding of landscape processes, but this is also related to the choice of the prior distribution of the scaling fingerprints. In line with Bayesian statistics, subjectivity plays a big role here. Different priors can be assumed to see their effect, and the priors can be eventually changed as additional information becomes available.

Very simple models have been used to derive the fingerprints for the case study. The change in the observed flood behavior is assumed to be driven by three components whereas the change in each component is represented by a single variable. For example, atmospheric changes are represented by observed changes in the maximum annual precipitation for a duration associated with catchment size. This assumption neglects a variety of other changes, such as changes in evapotranspiration or in the rainfall‐snow ratio. Similarly, it could be argued that the representation of river effects by a loss of retention volumes in the floodplains ignores flood‐moderating effects by dikes relocation, re‐naturalization of rivers, and implementation of polders along rivers. Against this background we stress the exemplary character of the case study. The assumptions need to be tailored to the dominant drivers of change in the specific region. For Upper Austria, we are confident that our assumptions, although very simple, represent the dominant changes during the study period. Further, the intention is to provide a first‐order analysis for the flood change attribution. The method is not limited to the setup used in this study. More complex, and possibly more realistic, changes could be introduced and state‐of‐the‐art atmospheric, hydrological, and hydraulic models could be used to derive prior information on the scaling fingerprints. There is room for additional work where these more complex cases are evaluated, in particular discussing the trade‐off between added model complexity and identifiability. One of the advantages of our approach in this context is that it allows determining how well the fingerprints need to be constrained to attribute flood changes. The scaling fingerprints can be estimated with uncertainty by modeling. If the uncertainty is too high, independent sources of information should be used to reduce it, such as past changes in catchment or river characteristics. Certainly, the proposed approach needs to be applied to different regions and extended by different conceptualizations of changes in the drivers in order to understand its potential and limitations.

Also note that the focus of the approach is the attribution of changes observed in the data in a given time window, rather than modeling the system for predictive purposes. The approach cannot be used directly for statements about the future, i.e., for prediction and design, since future changes in the drivers may be different from past ones (see *Koutsoyiannis and Montanari* [2015] for a discussion on predictive modeling of hydrologic change); however, it can inform the framing of possible future flood changes [*Sivapalan and Blöschl*, 2015].

While this paper exploits the idea that the fingerprints of different drivers scale differently with area, the proposed framework is more general and could be modified to take advantage of other driver‐effect linkages. Other candidate fingerprints could be seasonality, flood severity, or type of flood change. The underlying idea of the framework is that different drivers have different effects on flood characteristics. Climatic changes may modify flood seasonality, while catchment and river network changes may not. Land use may impact only small floods, while climate may affect small and large floods. Land use may lead to gradual flood changes, whereas river training may result in abrupt changes. This study analyses mean changes in flood magnitude, and it would be worthwhile to address changes in other flood characteristics, such as flood quantiles, within the proposed framework.

We believe that the framework proposed in this study is more rigorous than most of the current approaches for flood change attribution in that it uses regional information, it estimates quantitatively the contribution of several drivers to the observed change, and it assigns a quantitative confidence statement to the attribution. The extension from local attribution to the regional framework proposed here could constitute a similar shift in flood change attribution as the extension from local flood frequency to regional flood frequency analysis. Further, the framework is not limited to flood change, but could be applied as well to attribute other hydrological changes, e.g., in water availability or drought characteristics.

## Appendix A MCMC Algorithm Code

The following code represents the model

(A1) $$\frac{dY}{dt}+\varepsilon_{dY/dt}=\sum_{j}^{m}a_{j}S^{b_{j}},$$

where $\varepsilon_{dY/dt}∼N(0,\sigma_{dY/dt})$. The left‐hand side of equation (A1) represents observed trends and their uncertainty. The right‐hand side of equation (A1) represents the mixing model of *M* components with a scaling relationship with catchment area *S*.

data {

//n. of causes/drivers:

int<lower=1> M;

//n. of catchments:

int<lower=M> N;

//observed trends:

real dYdt[N];

//uncertainty in the observed trends:

real<lower=0> sigma_dYdt[N];

//normalised catchment areas:

real<lower=0> S[N];

//information on scaling fingerprints:

real mu_b[M]; real<lower=0> sd_b[M];

}

parameters {

real<lower=0> a[M];

real standard_b[M];

}

transformed parameters {

real b[M];

for (j in 1:M) {

b[j] <‐ mu_b[j] + standard_b[j]*sd_b[j];

}

}

model {

//regional expectation of trend:

real regexp_dYdt;

//improper uniform prior for a,

//defined for positive values only.

//b has a normally distributed prior:

for (j in 1:M) {

standard_b[j] ∼ normal(0, 1);

}

for (i in 1:N) {

regexp_dYdt <‐ 0;

for (j in 1:M) {

regexp_dYdt <‐ regexp_dYdt + a[j]*(S[i]^b[j]);

}

dYdt[i] ∼ normal(regexp_dYdt, sigma_dYdt[i]);

}

}

The code is run using Stan, a probabilistic programming language for specifying models in terms of probability distributions [*Stan Development Team*, 2015b]. For continuous parameters, Stan uses Hamiltonian Monte Carlo (HMC) sampling [*Duane et al*., 1987; *Neal*, 1994, 2011; *Stan Development Team*, 2015a], a form of Markov chain Monte Carlo (MCMC) sampling [*Metropolis et al*., 1953; *Robert and Casella*, 2004] that has the ability to converge more quickly to the target posterior distribution than the commonly used Metropolis‐Hastings algorithm. HMC combines random motion with molecular dynamics in order to generate efficiently new successive sampled states, which are less correlated than those produced by the Metropolis‐Hastings algorithm.

The MCMC algorithm is used to estimate the posterior distribution of the parameters *a_j_* and *b_j_* ( j=1,…,M) given the observed dY/dt and $\sigma_{dY/dt}$. The prior distribution for *a_j_* is assumed uniform over the entire positive real line (i.e., it is improper). The prior distribution for *b_j_* is assumed normal with mean $\mu_{b_{j}}$ and standard deviation $\sigma_{b_{j}}$ to be specified. Independence is assumed in the prior information between all *a_j_* and *b_j_* parameters.
